# Hydrophobia after Fourteen Month’s Incubation

**Published:** 1861-03

**Authors:** 


### Hydrophobia After Fourteen Months Incubation.

A case of hydrophobia is reported in the Dublin *Medical Press*, says the Philadelphia *Reporter*, in which "the usual symptoms of hydrophobia were presented, accompanied with an intense pain, which was referred to the *penis*. This came on in severe paroxysms, which made the patient struggle and scream. He had been bitten fourteen months previously by a dog which was said to have been rabid. The wound on his leg was at once cauterized, but it never healed, and became a chronic sore, which continued until the time of the attack. He died within twenty-four hours."

